# Evaluating the Single and Combined Effects of BMDM and PS Microplastics on *Chlorella* sp.: Physiological and Transcriptomic Insights

**DOI:** 10.3390/toxics13110946

**Published:** 2025-11-03

**Authors:** Jin Liu, Yankun Zhang, Fengyuan Chen, Dandan Duan, Xiaoping Diao

**Affiliations:** 1 Ministry of Education Key Laboratory for Ecology of Tropical Islands, College of Life Sciences, Hainan Normal University, Haikou 571158, China; 202411071300011@hainnu.edu.cn (J.L.);; 2 Shenzhen Key Laboratory of Marine Microbiome Engineering, Institute for Advanced Study, Shenzhen University, Shenzhen 518060, China; 3 State Key Laboratory of Marine Resources Utilization in South China Sea, Hainan University, Haikou 570228, China

**Keywords:** microplastics, butyl methoxydibenzoylmethane (BMDM), *Chlorella* sp., combined stress, transcriptomic

## Abstract

In the environment, the coexistence of microplastics (MPs) with other pollutants may either enhance or reduce the toxicity of MPs themselves or the co-occurring pollutants toward microalgae. This phenomenon is particularly notable when MPs interact with emerging pollutants, such as ultraviolet absorbers. This study investigates the single and combined exposure effects of ultraviolet absorber (Butyl methoxydibenzoylmethane, BMDM, 50 μg/L) and MPs (Polystyrene, PS, 10 mg/L, d = 1 μm) on *Chlorella* sp. with a stress duration of 7 days. The results showed that cell density, chlorophyll a (Chla) concentration, and physical properties of cell surface integrity were higher in the combined stress group compared to the BMDM single stress group. Furthermore, transcriptome sequencing analysis revealed that the number of differentially expressed genes (DEGs) in the combined exposure group (885 DEGs) was lower than in the single exposure groups (BMDM: 1870 DEGs and PS: 9109 DEGs). Transcriptomic profiling indicated that individual stressors of BMDM and PS disrupted 113 and 123 pathways, respectively, predominantly associated with protein synthesis and energy metabolism. Conversely, combined exposure significantly enriched 86 pathways, including ribosome function and oxidative phosphorylation, thereby manifesting an antagonistic effect. This study provides new insights into the effects of BMDM and PS on *Chlorella* sp. and offers valuable information for the risk assessment of multiple pollutants.

## 1. Introduction

Plastics are widely used due to their stability, lightweight, and low production cost [1]. However, the uncontrolled use of plastic products and ineffective management policies have resulted in plastic waste entering the ocean through various pathways, accounting for approximately 80% to 85% of marine litter [2,3]. Marine plastic waste degrades through physical, chemical, and biological processes, eventually transforming into microplastics [4]. Due to their small particle size and high specific surface area, microplastics readily adsorb hydrophobic organic pollutants in the marine environment, resulting in a form of combined pollution that may exacerbate toxic effects on aquatic organisms [5]. Studies have found that the combined effects of polystyrene (PS) and benzo[a]pyrene (B[a]P) significantly enhance the inhibition of *Chaetoceros muelleri* growth [6]. In contrast, other studies have pointed out that PS can reduce the toxicity of nonylphenol (NP) to microalgae (*Dictyosphaerium* sp.) [7]. Therefore, the internal mechanisms of the toxic effects of microplastics combined with other pollutants on marine microalgae are still unclear.

Ultraviolet (UV) absorbers are compounds that absorb UV radiation to protect the skin from UV harm [8]. Due to increasing public awareness and concern about the dangers of UV radiation, the demand for UV filters has risen [9]. Butyl methoxydibenzoylmethane (BMDM) is one of the most commonly used UV-A absorbers and has been detected in aquatic environments worldwide, with concentrations ranging from 10 to 1770 ng/L [10]. Due to its lipophilic nature, BMDM can accumulate in organisms over time. Peng et al. (2017) found that the highest concentrations of BMDM, up to 21 ng/g, were detected in species such as bighead carp, eels, and Japanese stone crabs [11]. Moreover, increasing evidence suggests that BMDM can cause developmental toxicity, reproductive toxicity, neurotoxicity, and endocrine disruption in organisms [12]. Microplastics readily adsorb BMDM in the marine environment, leading to combined pollution, which may pose significant threats to the health of organisms. Studies have found that both the PS single-stress group and the BMDM single-stress group promote energy reserves and food intake in *Daphnia magna*, while the combined stress inhibits food intake and growth, with a synergistic effect on the toxicity to *Daphnia magna* [13]. In contrast, other studies have found that PS can reduce the developmental toxicity of BMDM in *Danio rerio* embryos, exhibiting an antagonistic effect between the two [14]. Therefore, the toxicological mechanisms of the combined stress of BMDM and PS on organisms are under debate.

*Chlorella* sp. is a primary producer in marine ecosystems and is highly sensitive and vulnerable to environmental changes [15]. Additionally, as PS is a major component of plastic products in daily life, it is often used as a standard plastic type in toxicity studies and requires attention [1]. Human activities are frequent in coastal areas, leading to elevated concentrations of PS and BMDM in the marine environment, posing significant challenges to ecological systems [16,17]. However, few reports exist on the combined toxicity of these two pollutants on *Chlorella* sp.

Studies have indicated that benzophenone-type UV absorbers (BMDM) are widely distributed in polluted marine areas, with concentrations reaching hundreds of μg/L [18]. To investigate their toxic effects, laboratory studies often employ exposure concentrations as high as 1000 μg/L [19]. Therefore, considering both current environmental conditions and the objectives of this study, we conducted preliminary concentration-screening experiments and selected 50 μg/L of BMDM for stress exposure experiments on *Chlorella* to systematically assess its toxicity mechanisms and ecological risks. The identified 50 μg/L of BMDM concentration elicits measurable and biologically relevant responses without causing overwhelming acute toxicity, which could mask more subtle interactive effects. While using multiple concentrations would provide broader concentration-response insights, the selected BMDM levels were optimized to capture key interaction patterns (e.g., synergistic or antagonistic effects) under experimentally tractable and physiologically meaningful conditions. Additionally, the preliminary experiments on PS concentration screening revealed that PS concentrations ≥10 mg/L significantly inhibited *Chlorella* sp. growth, while concentrations ≤ 10 mg/L showed no significant effects or even promoted growth. Consequently, a PS concentration of 10 mg/L was adopted in subsequent experiments to eliminate its inherent interference and clarify the combined toxicity effects of BMDM and PS. Based on these considerations, the study selected PS with a diameter of 1 µm at a concentration of 10 mg/L and BMDM at a concentration of 50 µg/L to conduct a 7-day stress experiment on marine *Chlorella* sp. It aims to: (1) evaluate the effects of single and combined stress on the growth and physiological characteristics of *Chlorella* sp.; (2) elucidate the molecular mechanisms underlying the combined toxicity. By measuring physiological indicators such as cell density, cell surface morphology, and physical properties, photosynthetic pigment content, oxidative stress levels, and energy metabolism, the study reveals the combined toxic effects of PS and BMDM. Additionally, transcriptomic analysis was employed to preliminarily elucidate the molecular toxicological mechanisms. Although previous studies have explored the individual toxic effects of microplastics and organic pollutants on marine organisms, there is still a lack of systematic research on the specific impacts of combined exposure to these two types of pollutants on marine microalgae. Given the widespread presence of microplastics and organic pollutants in the marine environment and their potential combined toxicity, this study aims to systematically evaluate the toxic effects of combined exposure to PS and BMDM on *Chlorella* sp. through experimental approaches, in order to better understand the potential impacts of these pollutants on marine ecosystems. In addition, this study also employs transcriptomic analysis to preliminarily explore the molecular toxicological mechanisms of combined exposure to PS and BMDM, providing basic data and theoretical support for future research.

## 2. Materials and Methods

### 2.1. Chlorella *sp.* Culture and Reagents

The *Chlorella* sp. used in the experiment was purchased from Shanghai Guangyu Biotechnology (Shanghai, China). Polystyrene microplastics (PS, d = 1 μm) were obtained from Shanghai Guanbu Electromechanical Technology (Shanghai, China). The UV absorber standard BMDM (purity > 99%) was purchased from Aladdin Biochemical Technology (Shanghai, China).

The marine *Chlorella* sp. was cultured using f/2 medium with a pH of 8.5 and salinity of 35‰. The f/2 medium was prepared in filtered seawater supplemented with 75 mg/L NaNO_3_ and 5 mg/L NaH_2_PO_4_·H_2_O as macronutrients. Trace metals include EDTA-chelated iron (5.9 mg/L FeCl_3_·6H_2_O and 8.7 mg/L Na_2_EDTA·2H_2_O), along with manganese, zinc, cobalt, copper, and molybdenum at standard f/2 concentrations. Vitamins consisted of thiamine HCl (100 µg/L), biotin (0.5 µg/L), and cyanocobalamin (0.5 µg/L). All components were added from 1000 × concentrated stocks at 1 mL per liter of medium. Cultures were carried out in 250 mL Erlenmeyer flasks containing 100 mL of seawater. BMDM stock solution (1000 mg/L, dissolved in dimethyl sulfoxide, DMSO) and PS were added to the medium, with a solvent control (CK) consisting of DMSO alone, which was verified to have no effect on all treatments. *Chlorella* sp. in the logarithmic growth phase was inoculated into the medium with an initial cell density of 1.0 × 10^6^ cells/mL. The cultures were then incubated under laboratory conditions in a light incubator with a light/dark cycle of 12 h/12 h, a light intensity of 84 µmol photons m^−2^ s^−1^, and a temperature of 25 ± 0.1 °C. All media and related supplies used in the experiment were autoclaved at 121 °C for 30 min.

### 2.2. Exposure Experiment

In this study, the concentration of PS particles (d = 1 µm) was set at 10 mg/L. The concentration of BMDM was set at 50 µg/L. In the combined stress group, the PS concentration was 10 mg/L and the BMDM concentration was 50 µg/L, with DMSO-added *Chlorella* sp. solution used as the solvent control. The experimental culture period was 7 days, and samples were taken on day 7 to measure physiological indicators of *Chlorella* sp. growth during the experiment. Three replicate groups were set for each treatment, with the specific experimental design shown in Table 1.

### 2.3. Cell Growth and Photosynthetic Pigment Assays

Sampling was performed on the 7th day of *Chlorella* sp. cultivation. The procedure involved measuring cell density (cells/mL) using a hemocytometer (Marienfeld, Lauda-Königshofen, Germany) under an optical microscope (× 400).

*Chlorella* sp. samples collected after 7 days of exposure were used for photosynthetic pigment assays. The specific procedure is as follows: 2 mL of *Chlorella* sp. suspension was centrifuged at 2292 × *g* for 10 min to remove the supernatant. Then, 2 mL of 90% methanol was added, and the mixture was incubated overnight at 4 °C for chlorophyll extraction. After 24 h, the solution was centrifuged at 9168× *g* for 10 min, and the supernatant was collected for further analysis. The absorbance of the supernatant was measured using a UV-visible spectrophotometer (Shanghai Jinghua Technology Instrument, Shanghai, China) at 652 nm and 665 nm. The chlorophyll content was calculated using Equations (1) and (2).

Chla (mg/L) = 16.82 A_665_ − 9.28 A_652_ (1)

Chlb (mg/L) = 36.92 A_652_ − 16.54 A_665_ (2)

where Chla and Chlb represent chlorophyll a and chlorophyll b, and A_665_ and A_652_ represent the absorbance of the supernatant at wavelengths of 665 nm and 652 nm, respectively.

### 2.4. Assessment of PS and BMDM Interactive Effects on Chlorella *sp.*

The Abbott model, based on the independent action hypothesis (i.e., components in a mixture affect organisms through distinct mechanisms without interfering with each other), is commonly used to predict combined toxicity effects [20]. In this study, the individual toxicity values (growth inhibition rates) of PS (*T_PS_*) and BMDM (*T_BMDM_*) were first experimentally measured. Subsequently, the theoretical combined toxicity predicted value (*T_pre_*) was calculated using Formula (3).

(3) $$T_{Pre}=T_{PS}+T_{BMDM}-\frac{\left(T_{PS}\times T_{BMDM}\right)}{100}$$

where *T_pre_* represents the combined toxicity of PS and BMDM predicted by the Abbott model, while *T_PS_* and *T_BMDM_* denote the measured individual toxicity values of PS and BMDM, respectively.

By comparing the observed combined toxicity (*T_obs_*) with the predicted value (*T_pre_*), the ratio *T_obs_*/*T_pre_* can be used to determine the type of interaction between pollutants: a ratio of 1 indicates an additive effect; a ratio significantly greater than 1 indicates a synergistic effect; and a ratio significantly less than 1 indicates an antagonistic effect. This model regards any deviation from the theoretical prediction as evidence of interaction between pollutants, thereby providing a quantitative basis for elucidating the mechanism of combined stress effects of PS and BMDM.

### 2.5. Cell Morphology Observation and Physical Property Measurement

Scanning electron microscopy (SEM, Hitachi High-Tech, Tokyo, Japan) was used to observe the overall morphology of *Chlorella* sp. under different stress conditions. At the 7th day of exposure, *Chlorella* sp. was collected by centrifugation, fixed with 2.5% glutaraldehyde, and washed repeatedly with phosphate-buffered saline (PBS). The samples were then dehydrated using a series of graded ethanol solutions (30%, 50%, 70%, 80%, 90%, 95%, and 100%), followed by the addition of tert-butyl alcohol to fix the algal cells. After freeze-drying, the final morphology was observed.

Atomic force microscopy (AFM, Bruker, Billerica, MA, USA) was used to measure the mechanical properties of *Chlorella* sp. cells, such as surface viscosity and roughness modulus. The specific procedure was as follows: *Chlorella* sp. was collected by centrifugation, and the cells were washed twice with 3 mL of sterile water under low vacuum pressure (<50 mmHg) to prevent salt crystal formation during cell probing. Quantitative nanomechanical mapping mode was employed to measure the morphology and mechanical properties at room temperature and 50% relative humidity. The surface viscosity, roughness, and modulus of the cells were analyzed using Nano Scope Analysis 2.0 (USA).

### 2.6. Measurement of Oxidative Stress and Energy Metabolism Levels

After 7 days of exposure, *Chlorella* sp. biomass was accurately weighed, and a suspension was prepared by adding saline solution in a weight-to-volume ratio of 1:9. The mixture was homogenized at a low temperature (0–4 °C) and centrifuged at 573× *g* for 10 min, with the supernatant collected for analysis. Subsequently, total protein (TP), superoxide dismutase (SOD) Activity, catalase (CAT) activity, malondialdehyde (MDA) content, glutathione reductase (GR) activity, reduced glutathione (GSH) content, adenosine triphosphatase (ATPase) activity, and glucose (Glu) content were measured based on the methods from the Nanjing Jiancheng Bioengineering Institute (Najing, China) and briefly described as follows:

TP: Measured using a standard colorimetric method based on the reaction of proteins with Coomassie Brilliant Blue G-250 dye.

SOD activity: Assessed using a xanthine-xanthine oxidase system, where SOD activity is determined by its ability to inhibit the reduction of nitroblue tetrazolium (NBT) by superoxide radicals.

CAT activity: Determined by measuring the decomposition rate of hydrogen peroxide (H_2_O_2_) at 240 nm.

MDA content: Measured using a thiobarbituric acid (TBA) reaction, where MDA reacts with TBA to form a pink-colored complex that absorbs at 532 nm.

GR activity: Assessed by monitoring the reduction of oxidized glutathione (GSSG) to reduced glutathione (GSH) using NADPH as a cofactor.

GSH content: Determined using a colorimetric method based on the reaction of GSH with 5,5′-dithiobis-(2-nitrobenzoic acid) (DTNB) to form a yellow-colored complex that absorbs at 412 nm.

ATPase activity: Measured by monitoring the hydrolysis of ATP to ADP and inorganic phosphate, with the release of inorganic phosphate quantified colorimetrically.

Glu content: Determined using a glucose oxidase-peroxidase (GOPOD) method, where glucose is oxidized to gluconic acid and hydrogen peroxide, which then reacts with a chromogen to form a colored complex that absorbs at 505 nm.

### 2.7. Transcriptome Sequencing Analysis and qPCR Validation

*Chlorella* sp. samples collected after 7 days of exposure were used for transcriptome analysis. Total RNA was extracted using the Trizol (Invitrogen, CA, USA) method and quantified with a NanoPhotometer^®^ spectrophotometer. Additionally, RNA integrity was assessed using a Bioanalyzer (Agilent, CA, USA). Purified mRNA was used as a template to prepare cDNA fragments, which were separated by gel electrophoresis and used to construct a library. The cDNA library was sequenced on the Illumina Genome HiSeq™ 2500 platform (Illumina, CA, USA), and transcriptome assembly was performed. KEGG annotation was used for individual gene analysis, and KOBAS software 3.0 (China) was used to analyze the pathway enrichment of differentially expressed genes (DEGs).

To verify the reliability of the transcriptome results, qPCR was performed in this study. Six genes were selected from several key pathways identified in the enrichment analysis for gene expression level analysis. According to the instructions of the FastStart Essential DNA Green Master Mix (Roche, Switzerland) kit, 18s rRNA was used as the reference gene (with similar amplification efficiency to the target gene). Relative gene expression was quantified using the 2^−ΔΔCt^ method. The primers used for fluorescence quantitative PCR are listed in Table A1.

### 2.8. Data Statistical Analysis

Data were processed and plotted using Excel 2010 (Microsoft Corporation, Redmond, WA, USA) and GraphPad Prism 8.3.0 (GraphPad Software, San Diego, CA, USA). One-way analysis of variance (ANOVA) and Duncan’s post hoc test are performed for significance analysis of different treatments using SPSS 23.0 (IBM Corporation, Armonk, NY, USA). Data are expressed as mean ± standard error of the mean (Mean ± SEM), and significant differences between groups are indicated by different letters (*p* < 0.05).

## 3. Results

### 3.1. The Single and Combined Exposure Effects of MPs and BMDM on Chlorella *sp.* Growth

**Figure 1:**
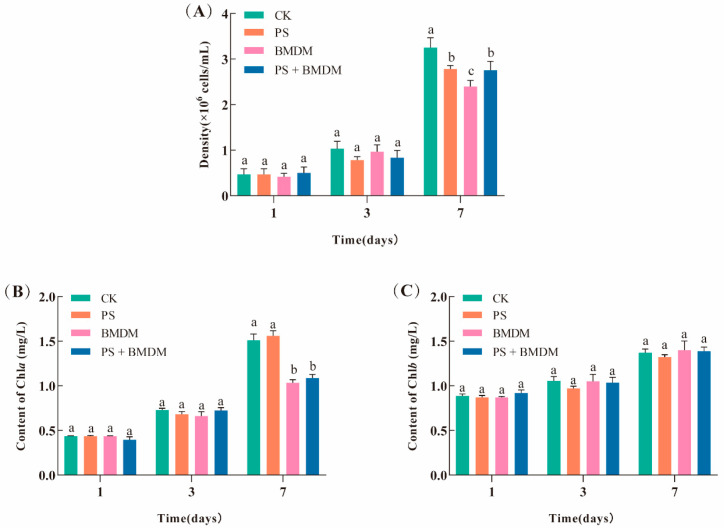
Effects of BMDM and PS on the growth and photosynthesis in *Chlorella* sp.: (**A**) cell density; (**B**) Chla content; (**C**) Chlb content. Note: CK: Solvent control (0.005% DMSO); PS: PS single exposure group (10 mg/L); BMDM: BMDM single exposure group (50 μg/L); BMDM + PS: Combined exposure group (50 μg/L BMDM + 10 mg/L PS). All measurements were performed in triplicate, and data are expressed as mean ± SEM. Significant differences between groups are indicated by different lowercase letters.

The *Chlorella* sp. density and the photosynthetic pigment content of different exposure groups at various time points were measured. The results indicated that there were no significant differences in algal density and photosynthetic pigment content among the groups after 1 and 3 days of exposure (*p* > 0.05). However, after 7 days of exposure, the algal density in the PS, BMDM, and combined exposure groups was significantly lower than that in the CK group (*p* < 0.05). Furthermore, compared with the control group, the Chla content in the BMDM exposure group and the combined exposure group decreased by 31.51% and 27.99%, respectively, and these differences were statistically significant (*p* < 0.05). (Figure 1) These findings suggest the potential adverse effects of prolonged exposure to PS and BMDM on the growth and photosynthesis of *Chlorella* sp.

According to the joint toxicity assessment results based on the Abbott model (Table 2), the *T_obs_*/*T_pre_* ratio for the combined stress group of PS and BMDM is 0.476 ± 0.039 (mean ± standard error), which is lower than the theoretical threshold of 1 under the assumption of independent action, indicating that the interaction between the two exhibits an antagonistic effect on *Chlorella* sp. toxicity.

Toxic effects of metal mixture were measured experimentally (*T_obs_*) and were predicted using Abbot’s model (*T_pre_*). *T_obs_*/*T_pre_* value = 1 indicated additivity of PS and BMDM individual effects; value < 1 indicates antagonism.

### 3.2. Chlorella *sp.* Cell Surface Morphology and Mechanical Properties

**Figure 2:**
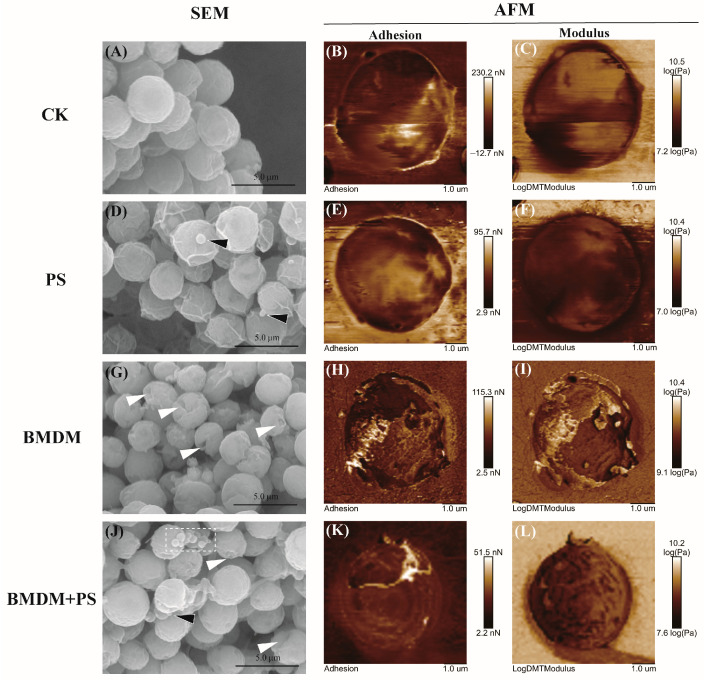
Effects of BMDM and PS on the morphology of *Chlorella* sp. cells. (**A**–**C**) Solvent control group; (**D**–**F**) PS stress group; (**G**–**I**) BMDM stress group; (**J**–**L**) combined stress group for cell morphology of *Chlorella* sp. The white dotted box indicates the aggregation of PS; a white arrow indicates damaged *Chlorella* sp.; the black arrows indicate PS adsorbed on *Chlorella* sp.

Single and combined exposure of BMDM and PS affected the cell morphology of *Chlorella* sp. (Figure 2). In detail, the cells exhibited intact cell walls, with relatively smooth surfaces and no noticeable indentation after 7 days in the control group (CK) (Figure 2A). As for the single PS stress group, a number of PS adhered to the surface of the *Chlorella* sp. cells, causing slight shrinkage and wrinkling, but no obvious changes were observed in the cell wall, and the cell morphology did not show severe damage (Figure 2D). On the contrary, after 7 days of single BMDM exposure, the surface of the *Chlorella* sp. cells exhibited varying degrees of wrinkling and indentation, leading to an apparent change in cell morphology (Figure 2G). Compared to the BMDM single stress group, the degree of wrinkling and indentation on the surface of the algal cells in the combined stress group was reduced in the PS and BMDM exposure group (Figure 2J).

The single and combined stress of BMDM and PS both caused changes in the physical properties of *Chlorella* sp. cell walls. In the control group, the adhesion and modulus values of the cell wall were relatively high, indicating normal mechanical strength and elasticity (Figure 2B,C), which are characteristics of healthy cells, as shown in Figure 2A. Under single PS stress, the adhesion and modulus values decreased (Figure 2E,F). In contrast, single BMDM stress resulted in a significant decrease in both adhesion and modulus values (Figure 2H,I), suggesting more severe damage to the cell wall (Figure 2G). Under combined stress conditions, the adhesion and modulus values of the cell wall partially recovered (Figure 2K,L) compared to the single BMDM stress but remained lower than those of the control group. This indicated that PS alleviated, to some extent, the damage caused by BMDM to the cell wall.

After 7 days of combined BMDM and PS stress, the changes in the physical properties of *Chlorella* sp. cell surfaces are shown in Figure 2. The surface viscosity of the combined stress group was significantly lower than that of the single BMDM and PS groups (*p* < 0.05) (Table A2). Moreover, the surface roughness of the algal cells is relatively higher in the single BMDM and combined PS and BMDM stress groups, but no significant difference was observed (*p* > 0.05) (Table A2). Notably, both single and combined PS and BMDM stress led to a reduction in the surface modulus of *Chlorella* sp. Compared to the control group, the surface modulus in the combined stress group was significantly reduced by 23.75% (Table A2).

### 3.3. The Oxidative Stress and Energy Metabolism Levels of Chlorella *sp.*

**Figure 3:**
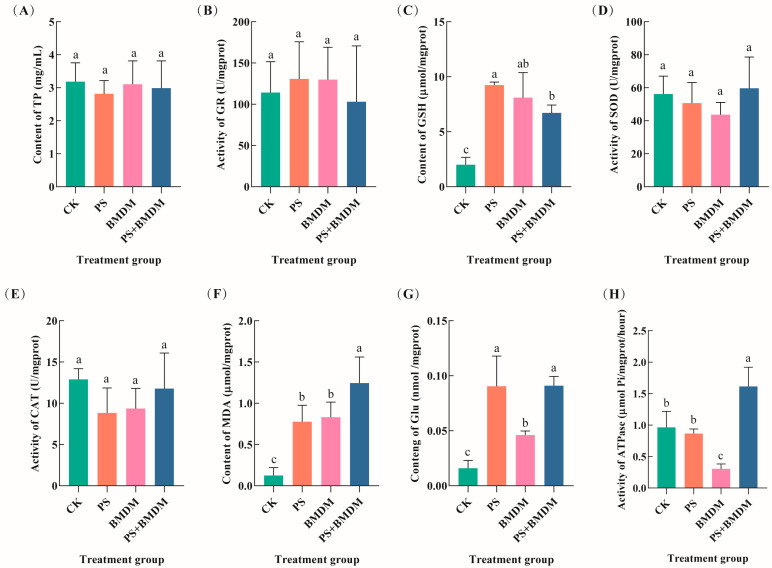
Effect of different concentrations of BMDM and PS on oxidative stress and energy metabolism indicators of *Chlorella* sp. (**A**) TP content; (**B**) GR activity; (**C**) GSH content; (**D**) SOD activity; (**E**) CAT activity; (**F**) MDA content; (**G**) ATPase activity; (**H**) glucose (Glu) content. Note: CK: Solvent control (0.005% DMSO); PS: PS single exposure group (10 mg/L); BMDM: BMDM single exposure group (50 μg/L); BMDM + PS: Combined exposure group (50 μg/L BMDM + 10 mg/L PS). All measurements were performed in triplicate, and data are expressed as mean ± SEM. Significant differences between groups are indicated by different lowercase letters.

Single and combined exposure to BMDM and PS can induce oxidative stress responses in *Chlorella* sp. There were no significant effects of single and combined BMDM and PS exposure on the TP content and GR activity in the algal cells (*p* > 0.05) (Figure 3A,B). However, single and combined exposure to BMDM and PS significantly increased the GSH content in *Chlorella* sp. (*p* < 0.05), with the combined exposure group showing significantly lower GSH levels than the PS exposure group (*p* < 0.05). Meanwhile, the GSH content in the combined exposure group was also lower than that in the BMDM exposure group (*p* > 0.05) (Figure 3C). No significant effects of single and combined exposure to BMDM and PS were observed on SOD and CAT activities (*p* > 0.05) (Figure 3D,E). Notably, both single and combined exposures to BMDM and PS significantly increased the MDA content in *Chlorella* sp. (*p* < 0.05) (Figure 3F).

The combined exposure to BMDM and PS also has effects on the energy metabolism of *Chlorella* sp. The results showed that the single PS exposure has no significant effect on *Chlorella* sp. ATPase activity (*p* > 0.05), while BMDM exposure decreases ATPase activity (*p* < 0.05). However, the combined exposure group showed a significant increase in ATPase activity (*p* < 0.05) (Figure 3G). Additionally, the single PS exposure had no significant effect on the Glu content in *Chlorella* sp. (*p* > 0.05), while BMDM exposure significantly decreased the Glu content (*p* < 0.05). Moreover, the Glu content in the combined exposure group was significantly higher than 67.55% (*p* < 0.05) (Figure 3H).

### 3.4. RNA-Seq Analysis and Differential Expression Genes (DEGs)

**Figure 4:**
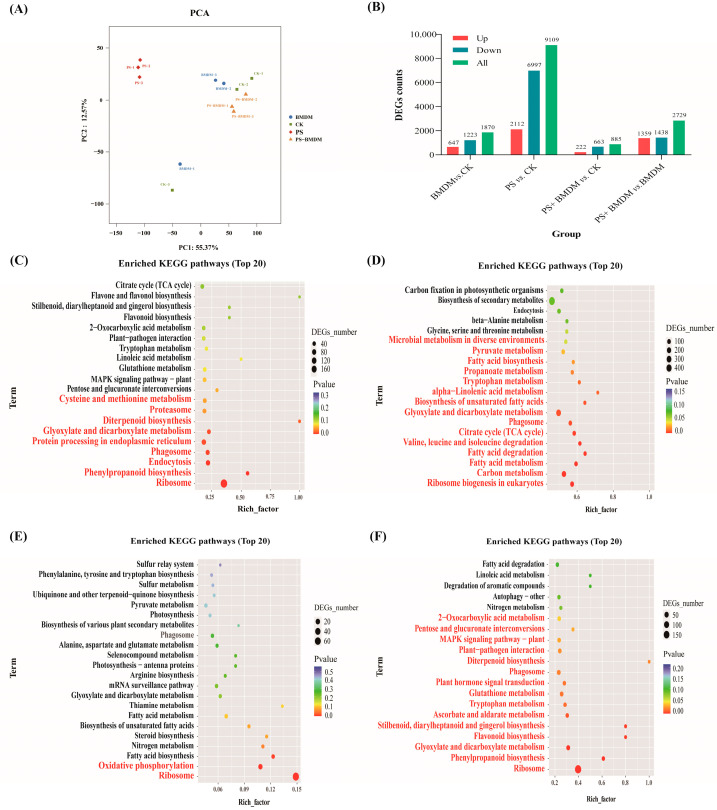
DEGs analysis and global transcriptome changes in *Chlorella* sp. under BMDM and PS exposure. (**A**) PCA; (**B**) histogram of DEGs; (**C**–**F**) KEGG enrichment analysis: (**C**) BMDM vs. CK; (**D**) PS vs. CK; (**E**) PS + BMDM vs. CK; (**F**) PS + BMDM vs. BMDM.

Principal component analysis (PCA) results (Figure 4A) showed a clear separation among the different treatment groups along the PC1 and PC2 dimensions. Notably, the combined stress group was positioned between the PS and BMDM single stress groups on the coordinate axes, displaying a transitional distribution pattern. This suggests that the combined stress of PS and BMDM may have an antagonistic effect on *Chlorella* sp., which is consistent with the earlier toxicity assessment results.

DEGs were screened between the CK group and other exposure groups. Compared to the solvent control group, a total of 1870 DEGs were identified in the BMDM group, with 647 DEGs upregulated and 1223 DEGs downregulated. In the PS group, 9109 DEGs were identified, with 2112 upregulated and 6997 downregulated. In the combined exposure group, 885 DEGs were identified, with 222 upregulated and 663 downregulated. When comparing DEGs between the BMDM and combined exposure groups, a total of 2797 DEGs were identified in the combined exposure group, with 1359 upregulated and 1438 downregulated (Figure 4B).

In this study, Kyoto Encyclopedia of Genes and Genomes (KEGG) enrichment analysis was performed on the selected DEGs, and the results showed that in the comparison of BMDM vs. CK, significant enrichment pathways (*p* < 0.05) included endocytosis, phagosome, ribosome, protein processing in the endoplasmic reticulum, proteasome, phenylpropanoid biosynthesis, glycolate and dicarboxylate metabolism, diterpenoid biosynthesis, and cysteine and methionine metabolism (Figure 4C).

In the comparison of PS vs. CK, significant enrichment pathways (*p* < 0.05) included phagosome, ribosome biogenesis in eukaryotes, fatty acid degradation, biosynthesis of unsaturated fatty acids, α-linolenic acid metabolism, fatty acid biosynthesis, carbon metabolism, fatty acid metabolism, citric acid cycle (TCA cycle), pyruvate metabolism, glycolate and dicarboxylate metabolism, valine, leucine, and isoleucine degradation, as well as glycine, serine, and threonine metabolism (Figure 4D).

In the comparison of BMDM + PS vs. CK, a total of 86 pathways were enriched, with only two pathways—ribosome and oxidative phosphorylation—showing significant enrichment (*p* < 0.05) (Figure 4E).

In the comparison of BMDM + PS vs. BMDM, significantly enriched pathways (*p* < 0.05) mainly include: phagosome, ribosome, plant hormone signal transduction, MAPK signaling pathway-plant, diterpenoid biosynthesis, glutathione metabolism, 2-oxocarboxylic acid metabolism, plant-pathogen interaction, aldehyde acid and dicarboxylate metabolism, ascorbate and aldarate metabolism, interconversion of pentose and glucuronic acid, phenylpropanoid biosynthesis, flavonoid biosynthesis, biosynthesis of diphenyl compounds, diarylheptanoids, and gingerol, as well as tryptophan metabolism (Figure 4F).

### 3.5. qPCR Validation

**Figure 5:**
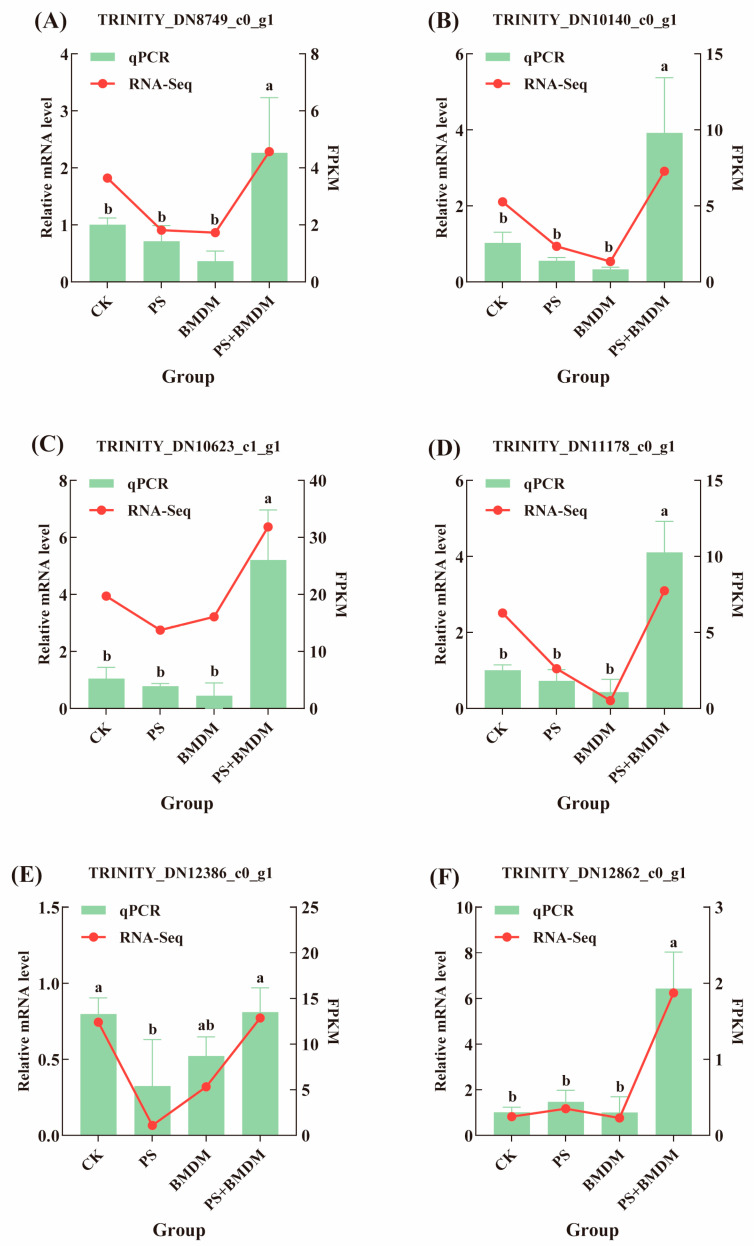
RT-PCR validations of DEGs. (**A**) TRINITY_DN8749_c0_g1; (**B**) TRINITY_DN10140_c0_g1; (**C**) TRINITY_DN10623_c1_g1; (**D**) TRINITY_DN11178_c0_g1; (**E**) TRINITY_DN12386_c0_g1; (**F**) TRINITY_DN12862_c0_g1.

To verify the reliability of the transcriptome sequencing results, we randomly selected six differentially expressed genes for qPCR analysis. These genes are involved in various key metabolic pathways, including protein synthesis, energy metabolism, and cell growth. The qPCR results were highly consistent with the transcriptome sequencing results, further confirming the reliability of the transcriptome data (Figure 5).

## 4. Discussion

### 4.1. Single and Combined Exposure of BMDM and PS Disrupted the Growth of Chlorella *sp.*

This study found that both single and combined exposures of BMDM and PS inhibited the growth of *Chlorella* sp. after 7 days. Notably, the cell density of Chlorella sp. in the combined stress group was higher than that in the single BMDM stress group, indicating that the antagonistic toxic effects of BMDM and PS on *Chlorella* sp. Additionally, Other studies have shown that the combined stress of PS and nonylphenol (NP) increased algal cell density, pigment concentration, and improved intracellular structure integrity, suggesting that PS can reduce NP toxicity to *Dictyosphaerium* sp. [7]. It is worth noting that some studies have also found that PS microplastics can completely counteract the inhibitory effects of 0.5 mg/L dibutyl phthalate (DBP) on *Chlorella pyrenoidosa*, where PS and DBP exhibited antagonistic effects [21]. The presence of PS reduced the toxicity of BMDM to *Chlorella* sp., and this effect may not only be due to the strong adsorption of small PS particles but also because *Chlorella* sp. activated its self-regulation mechanisms in response to external pressure, promoting internal damage repair and resisting external invasion.

The transcriptome results indicated that the number of differentially expressed genes (DEGs) in the combined stress group was lower than that in the BMDM and PS stress groups, suggesting that the single toxicities of BMDM and PS were greater than their combined toxicity. KEGG enrichment analysis revealed that single BMDM exposure affected metabolic pathways related to protein synthesis and degradation in *Chlorella* sp., such as ribosomes, protein processing in the endoplasmic reticulum, and proteasomes (Figure 4B). These pathways are typically closely associated with cell proliferation, growth, and protein synthesis [22]. Cell growth rates are often aligned with ribosome biosynthesis, and their expression is regulated to balance protein production in response to environmental changes [23,24]. Researchers have found that co-exposure to PS and perfluorooctanoic acid (PFOA) can disrupt the ribosomal pathway in *Chlorella sorokiniana*, triggering ribosomal stress and abnormal ribosome biosynthesis, leading to cell cycle arrest or apoptosis, thereby reducing algal cell proliferation [25]. This study also found that the single BMDM stress and combined stress affected the ribosomal pathway in *Chlorella* sp. (Figure 4B), suggesting that BMDM and combined stress may inhibit *Chlorella* sp. growth by affecting the ribosomal pathway. Additionally, the upregulation of the protein processing pathway in the endoplasmic reticulum (ER) helps increase the input of proteins into the ER, promote the output of properly folded proteins, and reduce the accumulation of misfolded proteins [26]. Meanwhile, the proteasome is the primary pathway for intracellular protein degradation [27]. This study observed significant enrichment of the ER protein processing and proteasome pathways in the BMDM exposure group (Figure 4B), indicating that BMDM might disrupt the tertiary structure of proteins by affecting the protein processing capacity of the ER. Furthermore, the inhibition of proteasome degradation functions could lead to the accumulation of misfolded proteins and induce apoptosis. Therefore, BMDM may inhibit *Chlorella* sp. growth by interfering with the processes of protein synthesis and degradation. It is noteworthy that the ER protein processing and proteasome pathways were not enriched in the combined stress group (Figure 4D), indicating that the inhibitory effect on *Chlorella* sp. growth in the combined stress group was lower than that in the BMDM group, consistent with the physiological measurements (Figure 1).

### 4.2. PS Reduced the Impacts of BMDM on the Morphological Structure of Chlorella *sp.*

According to the SEM results, single and combined exposure to BMDM and PS caused physical damage to *Chlorella* sp. cells. Previous studies have shown that microplastics can wrap around algal cells, limiting light absorption efficiency and hindering the exchange of substances with the external environment, which may negatively impact algal growth [28]. Notably, compared to the BMDM exposure group (Figure 2G–I), the degree of wrinkling on the surface of *Chlorella* sp., along with the adhesion and DMT Modulus in the combined exposure group, decreased (Figure 2J–L), and the number of indentations was reduced. This may be due to PS adsorbing BMDM from the medium, thereby reducing the chance of *Chlorella* sp. coming into contact with BMDM. On the other hand, heterogeneous aggregates of PS and BMDM could also adhere to *Chlorella* sp. cells, settling at the bottom of the medium and reducing the contact between newly divided *Chlorella* sp. cells and BMDM or PS, allowing better growth [29]. Furthermore, both individual exposure to BMDM and PS, as well as their combined exposure, led to a significant increase in the modulus (*p* < 0.05) and a notable decrease in adhesion (*p* < 0.05) of *Chlorella* sp. cells (Table A2). This suggests that the presence of PS may have a mitigating effect on the adverse impacts of BMDM. The increase in *Chlorella* sp. viscosity is typically associated with the amount of extracellular polymeric substances (EPS) on the cell surface, which serve as the first line of defense for algal cells [30,31]. When algal cells are exposed to nanoparticles or other exogenous toxic substances, they increase the secretion of EPS to resist external substances and defend themselves [32,33]. Compared with the single BMDM exposure group, the reduced surface viscosity of Chlorella sp. in the combined exposure group suggests (Figure 2E) that the damage caused by combined exposure is lower than that of BMDM exposure alone. In addition to viscosity, both single and combined exposure of BMDM and PS also affected the surface roughness of *Chlorella* sp. (Figure 2F). Generally, lower roughness indicates better cell wall integrity, and in the combined exposure group, *Chlorella* sp. exhibited lower roughness than in the BMDM exposure group, suggesting that *Chlorella* sp. is more susceptible to physical damage under BMDM exposure. The presence of PS may reduce BMDM’s attack on *Chlorella* sp., thereby providing a protective effect. Modulus represents the hardness of the cell surface, and environmental stress can induce changes in cell hardness in response to pollutants [34]. In this study, it was found that the surface modulus of *Chlorella* sp. in the combined exposure group significantly decreased (*p* < 0.05) compared to the other groups. Misic Radic et al. [35] found that the stress from nanoplastics can significantly reduce the hardness of microalgal cell walls, possibly due to the adsorption of nanoplastics on the microalgal surface. Thus, the addition of PS may adsorb to algal cells and decrease the hardness of the cell wall [35].

### 4.3. PS Mitigated the Inhibitory Effect of BMDM on the Photosynthesis of Chlorella *sp.*

The content of pigments reflects the changes in the algal ability to utilize light energy and is closely related to algal growth [36]. This study found that the BMDM stress significantly reduced the concentration of Chla in *Chlorella* sp., indicating that BMDM can inhibit chlorophyll synthesis (Figure 1B). This may be due to the attachment of BMDM on the surface of *Chlorella* sp., blocking light absorption and creating a shading effect, thereby affecting the photosynthetic process of *Chlorella* sp. (Figure 1B,C). This finding aligns with previous research showing that BP-3 inhibits the Chla content in Microcystis aeruginosa [37]. Notably, in the combined stress group, the addition of PS mitigated the inhibitory effect of BMDM on the *Chlorella* sp., possibly due to the adsorption of BMDM by PS in the medium, reducing the likelihood of contact between BMDM and *Chlorella* sp., similar to the result in the study where PS reduced the inhibitory effect of NP on the photosynthetic pigments of *Ctyosphaerium* sp. [7]. Additionally, it is found that PS alleviated the inhibitory effect of petroleum on Chla content, thereby increasing the photosynthetic rate of microalgae [38]. Other researchers have also noted that PS forms heavier heterogeneous aggregates with microalgae and settles to the water bottom, thus reducing the NP toxicity to the suspended algae cells [39].

Transcriptome results revealed that pathways such as tryptophan metabolism, ribosome biogenesis in eukaryotes, carbon fixation in photosynthetic organisms, photosynthesis-antenna proteins, and photosynthesis were enriched (Figure 4B–E). These pathways are closely related to fluctuations in *Chlorella* sp. growth and photosynthesis [40]. The results showed that although BMDM mainly affects diterpene biosynthesis and tryptophan metabolism, and PS affects tryptophan metabolism and carbon fixation pathways, single stress did not directly disrupt the photosynthesis pathway. However, the photosynthesis pathway was significantly disrupted in the combined stress group, suggesting that the BMDM and PS stress had a greater impact on photosynthesis than the single stress. Furthermore, pollutant exposure has been shown to reduce the PSII electron transport rate in Scenedesmus obliquus, leading to electron accumulation, enhanced photoinhibition, and increased Reactive Oxygen Species (ROS) production [41]. Excessive accumulation of ROS can damage cellular structures, block chlorophyll synthesis, and ultimately significantly reduce photosynthetic output while increasing toxicity [42].

### 4.4. PS Declined the Inhibitory Effect of BMDM on Chlorella *sp.* Energy Metabolism

ATPase is an important enzyme that primarily plays a role in material transport and energy conversion across the cell membrane. This study found that BMDM stress reduced the ATPase activity in *Chlorella* sp. cells, promoting the accumulation of Glu content, indicating that BMDM’s inhibitory effect on *Chlorella* sp. may involve disruption of energy metabolism, which is similar to the effects observed in *Chlorella pyrenoidosa* exposed to titanium dioxide nanoparticles (n-TiO_2_) [43]. The results showed that the ATPase activity in the combined stress group was significantly elevated, suggesting that PS could partially alleviate the inhibitory effect of BMDM on *Chlorella* sp. energy metabolism.

Transcriptomic results also indicated that the single BMDM exposure affected several energy metabolism-related pathways in *Chlorella* sp., such as endocytosis, aldehyde acid ester and dicarboxylic acid metabolism, pentose and glucuronic acid interconversion, 2-oxocarboxylic acid metabolism, and the citric acid cycle (TCA cycle) (Figure 4B). Aldehyde acid ester and dicarboxylic acid metabolism are closely associated with energy production and the generation of reductive equivalents (such as NADH and NADPH), which play key roles in antioxidant defense in plants. Particularly under organic pollutant stress, aldehyde acid ester metabolism can convert fatty acids into carbohydrates, thus providing additional energy support [44]. The TCA cycle is also involved in the conversion of fatty acids into carbohydrates, helping plants perform oxidative defense under adverse conditions [45]. Carbohydrate metabolism provides the essential precursor molecules for energy production and cellular protection in organisms [46]. Therefore, BMDM stress may disrupt *Chlorella* sp. energy metabolism by inhibiting carbohydrate formation.

The PS similarly affected several energy metabolism-related pathways in *Chlorella* sp., including fatty acid metabolism, fatty acid degradation, the citric acid cycle (TCA cycle), propionate metabolism, pyruvate metabolism, aldehyde acid ester and dicarboxylic acid metabolism, and endocytosis (Figure 4C). Acetyl-CoA, produced through fatty acid metabolism, can directly enter the TCA cycle to provide energy for the cell [47]. Pyruvate metabolism plays a crucial role in regulating oxidative stress responses, helping to maintain cellular vitality [48]. Moreover, aldehyde acid ester and dicarboxylic acid metabolism are key pathways for cellular antioxidant defense [44]. Therefore, PS stress may disrupt *Chlorella* sp. energy metabolism, weaken the cell’s antioxidant capacity, and trigger oxidative stress responses, leading to cellular damage. Additionally, pyruvate can promote the interconversion of sugars, fats, and amino acids through acetyl-CoA and the TCA cycle, supporting normal metabolic functions in *Chlorella* sp. [48]. Under changing environmental conditions, *Chlorella* sp. may induce propionate metabolism pathways to cope with external stresses and maintain metabolic balance.

In the combined exposure group, we observed that several energy metabolism-related pathways were affected, including oxidative phosphorylation, fatty acid biosynthesis, aldehyde acid ester and dicarboxylic acid metabolism, and pyruvate metabolism (Figure 4D). Oxidative phosphorylation provides the majority of ATP necessary for the survival of higher animals and plants and plays a key role in maintaining metabolic homeostasis. ATP, as the direct energy source for life activities, is involved in *Chlorella* sp. photosynthesis, nutrient transport (such as active transport), and cell division processes [48]. Therefore, combined stress may inhibit *Chlorella* sp. growth by impacting energy metabolism pathways such as oxidative phosphorylation.

### 4.5. PS Alleviated the BMDM-Induced Damage on Chlorella *sp.*

Glutathione (GSH), an important indicator of oxidative stress, plays a crucial role in eliminating excess ROS and reducing the toxic effects of pollutants on algae [49]. The study found that, compared to the BMDM exposure group, GSH levels in *Chlorella* sp. under combined stress were significantly reduced, indicating that the addition of PS could decrease lipid peroxidation in *Chlorella* sp., thereby alleviating the toxic effects of BMDM. Previous studies have suggested that PS (10 mg/L) increased the bioavailability of PFOA by altering cell membrane permeability, which in turn affected the toxicity of PFOA to *Chlorella* sorokiniana (synergistic effect), and *Chlorella* sp. could mitigate the effects by adjusting its antioxidant system [25]. This is contrary to the results of the present study, possibly due to differences in the type of pollutants and exposure concentrations.

BMDM exposure disrupted the cysteine and methionine metabolism, as well as tryptophan metabolism pathways in *Chlorella* sp. (Figure 4B). Cysteine can be used for the synthesis of GSH [50], which in turn is involved in scavenging ROS, thus reducing oxidative stress in the organism [51]. Methionine serves as a general initiator of protein synthesis [50]. Tryptophan functions as a signaling molecule that enhances the photosynthetic activity of microalgae [52]. By enriching these pathways, *Chlorella* sp. cells under BMDM stress strengthened their ability to maintain cellular antioxidant capacity, which is critical for cell function and protection against BMDM damage [53]. This also indicates that BMDM may interfere with certain amino acid metabolic pathways, affecting *Chlorella* sp. antioxidant capacity and photosynthesis.

PS exposure also affected the oxidative stress-related pathways in *Chlorella* sp., including the biosynthesis of unsaturated fatty acids, α-linolenic acid metabolism, and β-alanine metabolism pathways (Figure 4C). Unsaturated fatty acids help maintain cell membrane structure and function, and their levels can reflect the degree of membrane damage [54]. Glycine and threonine can act as cell protectants, scavenging ROS and stabilizing plasma membranes [45]. β-alanine metabolism contributes to antioxidant capacity [45]. α-linolenic acid is closely related to the synthesis of endogenous substances that protect cells from oxidative stress [55]. The degradation of valine, leucine, and isoleucine is crucial for protein synthesis, redox balance, antioxidant defense, detoxification, and maintaining nitrogen homeostasis [45]. This suggests that PS exposure disrupted these processes, thereby inducing oxidative stress in *Chlorella* sp. In response to this stress, *Chlorella* sp. utilizes the aforementioned metabolic pathways (biosynthesis of unsaturated fatty acids, α-linolenic acid metabolism, and β-alanine) to maintain osmotic balance, scavenge reactive oxygen species (ROS), and control cellular damage, thereby enhancing the algal resistance to PS stress. Notably, serine is an essential substance for the biosynthesis of Chla, which is highly related to the photosynthesis system and the stimulated photosynthetic process [45]. Therefore, PS stress can interfere with *Chlorella* sp. photosynthesis and oxidative stress by affecting lipid metabolism and amino acid metabolism.

Combined stress also disrupted oxidative stress-related pathways in *Chlorella* sp., such as the biosynthesis of unsaturated fatty acids, thiamine metabolism, and arginine biosynthesis pathways (Figure 4D). Arginine has antioxidant properties and can act as a free radical scavenger, helping to neutralize harmful ROS and protect cells from oxidative stress related to metal exposure [45]. Combined stress also interfered with amino acid metabolism pathways in *Chlorella* sp., such as alanine, aspartate, and glutamate metabolism (Figure 4D). These metabolic pathways not only contribute to protein synthesis but also play key roles as intermediates in various metabolic processes [45].

Additionally, the phagosome pathway and glyoxylate and dicarboxylate metabolism pathways were significantly enriched under BMDM, PS, and combined stress conditions (Figure 4B–D). As a vesicular structure responsible for engulfing and degrading exogenous substances, the phagosome plays a crucial role in the cell’s immune and defense mechanisms. This may be due to the inhibition of gene expression related to pollutant internalization, thereby reducing the internalization of pollutants and protecting *Chlorella* sp. from damage caused by BMDM and PS. This could be one of *Chlorella* sp.’s defense mechanisms in response to environmental stress [27]. Reports have indicated that the EPS secreted by algal cells is associated with endocytosis, and the presence of EPS can significantly influence the endocytosis of TiO_2_ by microalgae. EPS acts as a barrier, reducing the internalization of nano-TiO_2_ mediated by related proteins [56]. Thus, *Chlorella* sp. may also protect itself from BMDM and PS exposure by secreting EPS to lower their concentrations in the culture medium.

The mitogen-activated protein kinase (MAPK) signaling pathway interacts with ethylene, auxin, jasmonic acid, abscisic acid, and phospholipid signaling pathways, playing a pivotal role in the stress response, signal transduction, and antioxidant activities in microalgae. It is often activated by external stimuli [57,58]. KEGG enrichment analysis revealed that the MAPK signaling pathway was significantly enriched under combined stress compared to BMDM stress alone. This indicates that combined stress enhances MAPK signaling in *Chlorella* sp., enabling it to quickly perceive environmental changes and activate protective mechanisms through gene regulation. Therefore, PS may alleviate BMDM-induced damage by enhancing MAPK signaling, accelerating environmental adaptation, and promoting stress-related gene expression.

## 5. Conclusions

This study investigated the toxic effects of BMDM and PS on marine *Chlorella* sp. and explored the molecular mechanisms underlying their combined stress. The results showed that BMDM and PS single disrupt metabolic pathways related to protein synthesis, energy metabolism, and oxidative stress in *Chlorella* sp., while combined stress further impairs photosynthetic pathways. Notably, an antagonistic interaction was observed, with PS alleviating the toxicity induced by BMDM. These findings reveal the combined toxicological effects of the organic pollutant BMDM and spherical microplastic PS on marine microalgae, providing new insights into their ecological impact.

## Figures and Tables

**Table 1:** Design of combined exposure experiments.

Group	Pollutants	Concentration
CK	DMSO	0.005%
PS Single toxicity	PS	10 mg/L
BMDM Single toxicity	BMDM	50 μg/L
Combined toxicity	BMDM + PS	50 μg/L + 10 mg/L

CK: Control check; DMSO: dimethyl sulfoxide; PS: polystyrene; BMDM: Butyl methoxydibenzoylmethane.

**Table 2:** Interactive effects of PS microplastics and butyl methoxydibenzoylmethane (BMDM) on *Chlorella* sp. cell density after 7 days of treatment.

Treatment	*T_obs_*	*T_pre_*	*T_obs_*/*T_pre_*
PS + BMDM	0.096 ± 0.225	0.178 ± 0.550	0.476 ± 0.039

## Data Availability

The original contributions presented in the study are included in the article. Further inquiries can be directed to the corresponding author.

## Appendix A

### Appendix A.1

**Table A1:** Primer sequences for qPCR.

Gene ID	Primer Sequences
TRINITY_DN10623_c1_g1	F: GGTGCAGTCGGTAAGACATGC
	R: TGACCTGCTGTATCCCATAAGGAG
TRINITY_DN11178_c0_g1	F: CCACTCAAGAAGTCCACCCATAAC
	R: AGTCAGAAACACACTCTACAAGGC
TRINITY_DN12386_c0_g1	F: CCGACAGTACCATCACCAACAAC
	R: TGCAGAGACCAGAACAAAGAAACG
TRINITY_DN12862_c0_g1	F: TTTTGGAGTTGGGGATCGTCATC
	R: AAATGGTGGTGGAAATGGTTTTGG
TRINITY_DN8749_c0_g1	F: GCATCAGCACCACCAACACC
	R: AGGATTCTGGAAACCTGGTAGTGG
TRINITY_DN10140_c0_g1	F: GGACCTGGAGTTGGAATTGATGG
	R: ACATGGTTGAGAATCAGCACAATC
18s rRNA	F: GAGTATGGTCGCAAGGCTGAA
	R: AACCTGACAAGGCAACCCAC

### Appendix A.2

**Table A2:** Effects of BMDM and PS on the physical properties of *Chlorella* sp. cells. Values are expressed as Mean ± SEM, *n* = 6.

Group	Roughness (nm)	Adhesion (nN)	Modulus (MPa)
CK	8.715 ± 2.060 ^a^	44.917 ± 3.496 ^a^	11209.833 ± 1914.712 ^a^
PS	9.333 ± 2.092 ^a^	48.567 ± 8.859 ^a^	10724.500 ± 1227.719 ^a^
BMDM	11.422 ± 2.621 ^a^	44.900 ± 7.186 ^a^	10175.333 ± 617.196 ^a^
BMDM + PS	11.052 ± 1.939 ^a^	36.35 ± 5.440 ^b^	7759.000 ± 2202.216 ^b^

Note: The same letter indicates no significant difference (*p* > 0.05), while different letters indicate significant differences (*p* < 0.05).
